# Modeling specific aneuploidies: from karyotype manipulations to biological insights

**DOI:** 10.1007/s10577-023-09735-7

**Published:** 2023-08-29

**Authors:** My Anh Truong, Paula Cané-Gasull, Susanne M. A. Lens

**Affiliations:** Oncode Institute and Center for Molecular Medicine, University Medical Center Utrecht, Universiteitsweg 100, 3584 CG Utrecht, The Netherlands

**Keywords:** Aneuploidy, CIN, chromosome, cancer, CRISPR/Cas9

## Abstract

An abnormal chromosome number, or aneuploidy, underlies developmental disorders and is a common feature of cancer, with different cancer types exhibiting distinct patterns of chromosomal gains and losses. To understand how specific aneuploidies emerge in certain tissues and how they contribute to disease development, various methods have been developed to alter the karyotype of mammalian cells and mice. In this review, we provide an overview of both classic and novel strategies for inducing or selecting specific chromosomal gains and losses in human and murine cell systems. We highlight how these customized aneuploidy models helped expanding our knowledge of the consequences of specific aneuploidies to (cancer) cell physiology.

**Supplementary Information:**

The online version contains supplementary material available at 10.1007/s10577-023-09735-7.

## Introduction

Aneuploidy, defined as a number of chromosomes that deviates from a multiple of the haploid genome, is a prominent feature of spontaneous pregnancy loss, congenital disorders such as Down syndrome, and of cancer (Hassold et al. 1980; Hassold and Jacobs 1984; Sahoo et al. 2017; Ben-David and Amon 2019; Gruhn and Hoffmann 2022). This karyotype aberration is one of the consequences of erroneous chromosome segregation during meiosis and mitosis (referred to as chromosomal instability, or CIN) and includes gains and losses of whole chromosomes or chromosomal arms. Long before the first genomic alterations in proto-oncogenes were discovered in human cancers (Der et al. 1982; Parada et al. 1982; Dalla-Favera et al. 1982), aneuploidy was already described as a distinct feature of cancer by Boveri (Boveri 2008, translated from the original 1914 article). In fact, abnormal karyotypes are observed in ~80% of all solid tumors and in ~60% of hematopoietic cancers (Weaver and Cleveland 2006; Duijf et al. 2013; Taylor et al. 2018). While the role of CIN in cancer initiation and metastasis is becoming increasingly evident (Bakhoum et al. 2018, reviewed in van Jaarsveld and Kops 2016; Sansregret et al. 2018; Tijhuis et al. 2019), the exact contribution of whole chromosome or chromosomal arm gains and losses to disease development has remained less clear (Ben-David and Amon 2019). This knowledge gap is in part due to the challenges associated with generating mammalian models with specific aneuploidies. In this review, we focus on various strategies developed over the years to manipulate the karyotypes of human and mouse cells and animals.

Of note, *Saccharomyces cerevisiae* (budding yeast) has been a valuable organism for modeling aneuploidies, thanks to its short doubling time, the possibility of karyotype manipulations through mating, and genetic tools readily applicable for this model organism. These yeast-specific methods have been reviewed elsewhere (Mulla et al. 2014; Gilchrist and Stelkens 2019), but it is worth highlighting that they have produced valuable collections of stable isogenic aneuploid strains. These strains have been used to uncover generic cellular responses to aneuploidy (Torres et al. 2007; Pavelka et al. 2010; Beach et al. 2017; Ravichandran et al. 2018). Subsequent analyses of human and mouse cell lines carrying specific trisomies or monosomies have revealed very similar effects on cell physiology as seen in yeast, including impaired cellular fitness and proliferative potential due to the proteotoxic, metabolic and replication stresses associated with chromosomal gains, or impaired ribosomal biogenesis linked to chromosomal losses (Williams et al. 2008; Stingele et al. 2012; Nicholson et al. 2015; Meena et al. 2015; Ohashi et al. 2015; Santaguida et al. 2015; Passerini et al. 2016; Passerini et al. 2016; Chunduri et al. 2021).

While the adverse effects of aneuploidy may explain its detrimental consequences for the developing embryo, it also raises the “aneuploidy paradox” in cancer. Why does a disease characterized by proliferation frequently exhibit aneuploid karyotypes? Furthermore, different tumor types display distinct aneuploidy patterns. For instance, colorectal tumors commonly have recurrent gains of chr7, 13, 20 and loss of chr18, while clear cell renal tumors commonly lose chr3p and gain chr5q (Knouse et al. 2017; Mitchell et al. 2018). How these cancer-specific aneuploidy patterns are established, and to what extent they contribute to disease progression remains a major puzzle in the field. Chromosome loss may support the loss of heterozygosity (LOH) of tumor suppressor genes, while chromosome gain could drive the amplification of one or more oncogenes (Nowak et al. 2002; Rajagopalan et al. 2003). Consistent with this idea, the presence of oncogenes or tumor suppressor genes on certain chromosomes correlates with their recurrent gain or loss in cancer (Davoli et al. 2013). Still, accomplishing LOH or oncogene amplification by losing or gaining an entire chromosome appears challenging because of the collateral deregulation of hundreds of other genes on the affected chromosome. An alternative hypothesis is that the gain or loss of specific chromosomes is tolerated in a tissue-specific manner. Chromosomes harboring genes that are highly expressed in certain tissues may be more easily tolerated as trisomy in those tissues because such gains would cause a milder gene expression imbalance (Sack et al. 2018; Patkar et al. 2021). While attractive, these hypotheses have never been formally tested, partly because it requires the modeling of specific aneuploidies across different tissue types. Finally, whether tumors are addicted to recurrent aneuploidies is another question currently addressed in the field (Girish et al. 2023). While certain cancers are dependent on an activated oncogene to maintain their malignant properties (Weinstein and Joe 2008), it is less clear how the elimination of specific recurrent aneuploidies from cancer cells affects cancer maintenance (Girish et al. 2023).

To understand the contribution of specific chromosomal gains or losses to developmental disorders and cancer, there is a clear need to create models with customized aneuploidies. We will discuss the current successful strategies to generate chromosome-specific gains and losses in mouse and human model systems. Each method offers unique opportunities and distinct hurdles in their experimental application. We will highlight the general principles of these approaches, focus on the insights into cancer biology thus far gathered through their application, and discuss future developments and prospects for the field.

## Strategies to introduce specific chromosomal gains

Many of the present mammalian cell lines and mouse strains that possess a single chromosome gain, or a corrected chromosomal loss have been generated using microcell-mediated chromosome transfer (MMCT) or by crossing mice with Robertsonian translocations. The models generated by these techniques provided significant insights into the effects of aneuploidy on non-transformed and cancer cell physiology, as well as developmental disorders like Down syndrome.

### Microcell-mediated chromosome transfer

**Fig. 1:**
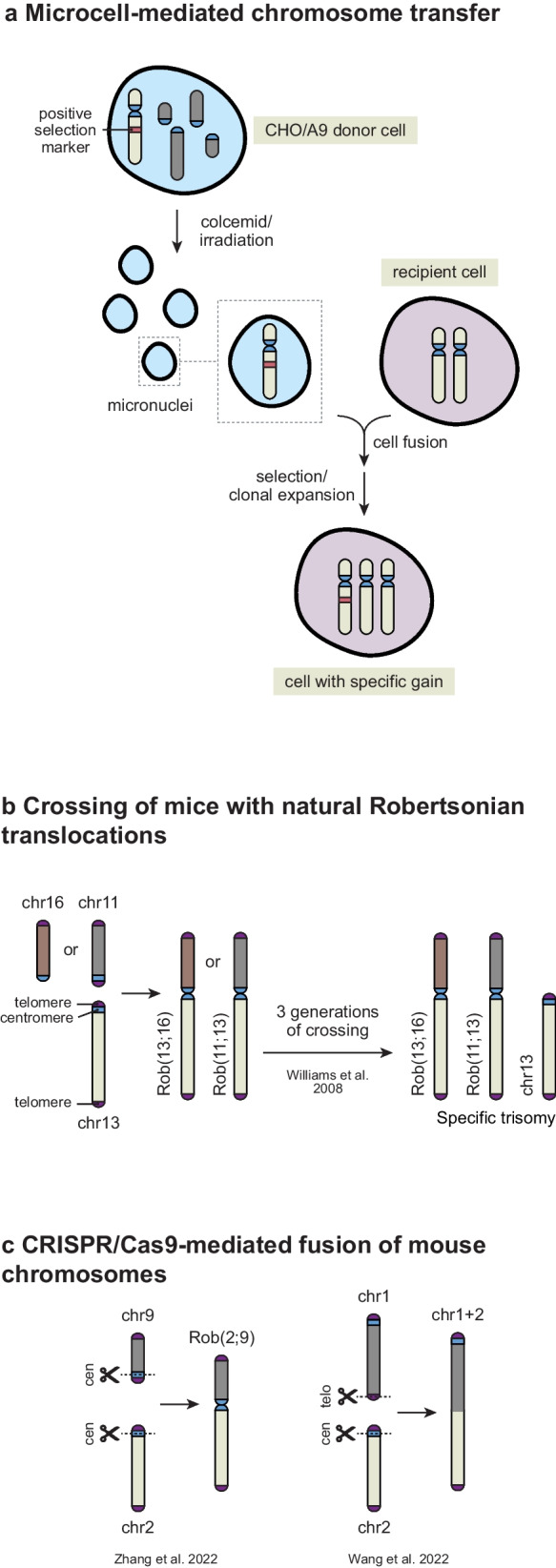
Strategies to introduce an additional chromosome into mammalian cells and mice. **a** Microcell-mediated chromosome transfer consists of 2 steps: (1) micronuclei formation in donor cells by colcemid treatment or irradiation; and (2) fusion of micronuclei to recipient cells of choice. Hybrid Chinese hamster ovary (CHO) or mouse A9 cells containing a single human chromosome are mostly used as donor cells (Tanabe et al. 2000). A positive selection marker, (often puromycin or neomycin resistance genes), is usually integrated into the chromosome of interest to facilitate the recovery of recipient cells with specific chromosomal gains. **b** Crossings of mice carrying Robertsonian chromosomes can generate mice and mouse ESCs with specific trisomies (Williams et al. 2008). As starting point, two mouse strains are crossed each carrying a different Robertsonian translocation involving the chromosome of interest (e.g., Rob (13;16) and Rob (11;13)). **c** CRISPR/Cas9-induced targeted chromosome fusions can generate either Robertsonian-like metacentric chromosomes (Zhang et al. 2022) or a large telocentric fused chromosome (Wang et al. 2022) (Supplemental Table 2). This could be applied to create parental mouse strains carrying specific (viable) chromosome fusions for crossings that eventually generate specific trisomies

MMCT was originally developed by Fournier and Ruddle in the 1970s as a method for introducing murine chromosomes into mouse, Chinese hamster, and human cells (Fournier and Ruddle 1977). The process involves two steps: first, the induction of micronucleation in donor cells via irradation or prolonged treatment with colcemid (i.e., a drug that depolymerizes microtubules), followed by a rate-limiting second step, in which the micronuclei are fused to recipient cells of choice (Fig. 1a). To facilitate subsequent positive selection of recipient cell clones with the specific chromosomal gain, an antibiotic resistance gene is usually integrated in the chromosome of interest from the donor cell line. The most commonly used donor cells for MMCT are mouse A9 fibroblasts and Chinese hamster ovary (CHO) cells, as these cells easily form micronuclei and can survive prolonged exposure to colcemid (Nakayama et al. 2015). The application of MMCT to human cell systems has been greatly improved by the generation and commercialization of libraries of CHO/human and A9/human hybrid donor cell lines, each containing a selectable single human autosome or chromosome X (Kugoh et al. 1999; Tanabe et al. 2000).

MMCT was originally used for biological applications ranging from gene mapping to gene imprinting analysis (Saxon et al. 1986; Kugoh et al. 1999; reviewed in Yoshida et al. 2000; Meaburn et al. 2005) and to model embryonic aneuploidies leading to congenital disorders (reviewed in Akutsu et al. 2020). These included the generation of mouse models for Down syndrome by transferring either a large part of human chr21 (Shinohara et al. 2001; O’Doherty et al. 2005), or a mouse artificial chomosome containing the q-arm of the human chr21, into mouse embryonic stem cells (ESCs) (Kazuki et al. 2020). Moreover, early studies in the 90s utilized MMCT to map tumor growth suppression and anti-metastatic activities on human chromosomes by reverting a chromosomal loss or inducing a specific chromosomal gain in various cancer cell lines (Oshimura et al. 1989; Yamada et al. 1990; Kugoh et al. 1990; Ogata et al. 1993; Tanaka et al. 1998). As a result, a considerable number of human, mouse, and rat cell lines that harbor an extra single human chromosome were generated (reviewed by Kugoh et al. 2016). Functional studies on these cell lines led to the hypothesis that loss of a specific chromosome or chromosome arm could be a strategy to silence tumor-suppressing genes located on that chromosome. For example, MMCT of human chr3 into 3p monosomic renal carcinoma cells induced senescence *in vitro* (Ohmura et al. 1995; Tanabe et al. 2000). Similarly, induction of trisomy for chr3p or the whole chr3 in oral squamous carcinoma cells inhibited cellular growth and suppressed tumor formation in athymic mice upon subcutaneous injection (Uzawa et al. 1995; Nishio et al. 2015). These anti-tumorigenic effects of chr3 gain were subsequently attributed to multiple tumor-suppressing genes on chr3p, including several telomerase repressors (Uzawa et al. 1998; Abe et al. 2010; Nishio et al. 2015). Furthermore, transferring either whole chromosomes or parts of human chromosomes 1-8, 10-13, 16-20, 22, X, or Y into different cancer or non-transformed immortalized cell lines mainly inhibited the proliferative, tumorigenic and/or metastatic potential of the recipient cell line (reviewed in Yoshida et al. 2000; Meaburn et al. 2005; and Kugoh et al. 2016). However, only in a few cases could the tumor-suppressing effects of a specific chromosome transfer be narrowed down to a single gene or gene cluster (Dong et al. 1995; Yoshida et al. 1999; Seraj et al. 2000; Goldberg et al. 2003).

More recent studies focused on the generic and specific effects of chromosomal gains on cell physiology. Analyses of transformed and non-transformed human cell lines with a single trisomic chromosome (Supplemental table 1) revealed that a chromosome gain often leads to increased CIN, replication stress, as well as global transcriptomic and proteomic changes (Phillips et al. 2001; Phillips et al. 2001; Nawata et al. 2011; Stingele et al. 2012; Dürrbaum et al. 2014; Nicholson et al. 2015; Passerini et al. 2016). Furthermore, in line with earlier observations (Yoshida et al. 2000; Meaburn et al. 2005; Kugoh et al. 2016), these recent studies also showed that nearly all single chromosomal gains negatively impact cellular transformation and metastasis formation. However, this generally appears not to be due to expression of tumor-suppressing genes on the gained chromosome, but rather due to the stresses associated with the gene expression imbalances (Vasudevan et al. 2020). A few studies reported chromosome-specific effects on human cell physiology. For instance, MMCT of chr2, but not of chromosomes 3, 8, 7, 11, or 12, induced senescence in human cervical cancer cells (Uejima et al. 1995). In contrast, introduction of an extra copy of chr7, but not of chromosomes 1, 2, 6, 9, or 11, was found to have tumor-suppressive effects in human choriocarcinoma cells CC1 (Matsuda et al. 1997). Moreover, trisomy of chromosome 8, 16, 17, or 19 suppressed metastatic behavior of human colorectal cancer HCT116 cancer cells, whereas trisomy of chr5 enhanced their metastatic potential (Vasudevan et al. 2020). Meanwhile, trisomy of chr7 and chr13 conferred a growth advantage to colorectal cancer DLD-1 cells when cultured under challenging conditions such as hypoxia or low serum, although these aneuploid cells proliferated slower than their euploid counterparts in standard culture conditions (Rutledge et al. 2016). Interestingly, trisomy 13, but not trisomy 7, was found to cause cytokinesis failure and elevated CIN in human colorectal cancer DLD-1 cells due to SPG20 overexpression (Nicholson et al. 2015). Despite these significant findings, their clinical relevance and the molecular mechanisms by which these aneuploidies potentially drive cancer progression and metastasis require further investigation.

### Robertsonian translocations

The second approach to generate specific trisomies takes advantage of Robertsonian fusions, which naturally occur in various races of the house mouse (M.m. domesticus). A translocation or fusion is considered Robertsonian when two different chromosomes are attached to each other at their centromeres (Fig. 1b; Robertson 1916). In mice, which have telocentric chromosomes, Robertsonian translocations result in the fusion of two entire chromosomes into one large metacentric chromosome. Although over a hundred races of M.m. domesticus with at least one Robertsonian metacentric chromosome have been identified in the wild, not all of these are used as laboratory mouse strains (Capanna et al. 1976; Garagna et al. 2014). By crossing mice according to a complex protocol, trisomic embryos for chr1, 13, 16, or 19 have been obtained within three generations (Supplemental table 1, Fig. 1b, Williams et al. 2008). However, none of these trisomic embryos survived to term, except for those with an extra copy of chr19, which lived for a short time after birth. All trisomic embryos exhibited developmental abnormalities such as growth restriction and nuchal edema, demonstrating the overall detrimental effects of a single chromosomal gain on mouse embryonic development. Additionally, studies using mouse embryonic fibroblasts (MEFs) derived from these trisomic embryos showed that having an extra chromosome led to metabolic changes, inhibited cell proliferation, and impeded oncogene-induced transformation (Williams et al. 2008; Sheltzer et al. 2017). While powerful, this method is only applicable to mice and cannot be used to study the contribution of a particular trisomy to cancer development, due to the limited lifespan of the trisomic animals and the inherent incompatibility of the method with conditional and tissue-specific control over the event that generates the trisomy. Moreover, it is difficult to apply to other chromosomes since the combinations of Robertsonian chromosome fusions in laboratory mice are limited. However, this limitation may be overcome by recent advances that allow for targeted whole-chromosome fusions via CRISPR/Cas9-induced DNA double stranded breaks (DSBs) in telomere and centromere regions of different mouse chromosomes (Fig. 1c, Supplemental table 2). Thus far, this impressive tour de force has delivered viable homozygous pups carrying engineered fusion of chr4-5, chr1-13, chr2-11, and chr5-17 (Supplemental table 2; Wang et al. 2022; Zhang et al. 2022). In theory, these mice could be used to create mouse strains with different specific chromosomal gains using similar crossing protocols as those used for mice with natural Robertsonian fusions (Williams et al. 2008, Fig. 1b).

## Methods to eliminate specific chromosomes

A quarter of a century ago, Lewandoski and Martin pioneered the field of targeted chromosome elimination by generating male mice that lacked an entire copy of the Y chromosome (X0) (Lewandoski and Martin 1997) through the use of Cre recombinase and a male transgenic mouse line that had accidentally integrated inverted *loxP* sites into the Y chromosome. With the introduction of targetable nucleases such as CRISPR/Cas9 and TALEN, the toolbox for targeted chromosome elimination has greatly expanded, allowing for more efficient manipulation of karyotypes.

### Cre/*loxP*-mediated chromosome loss

**Fig. 2:**
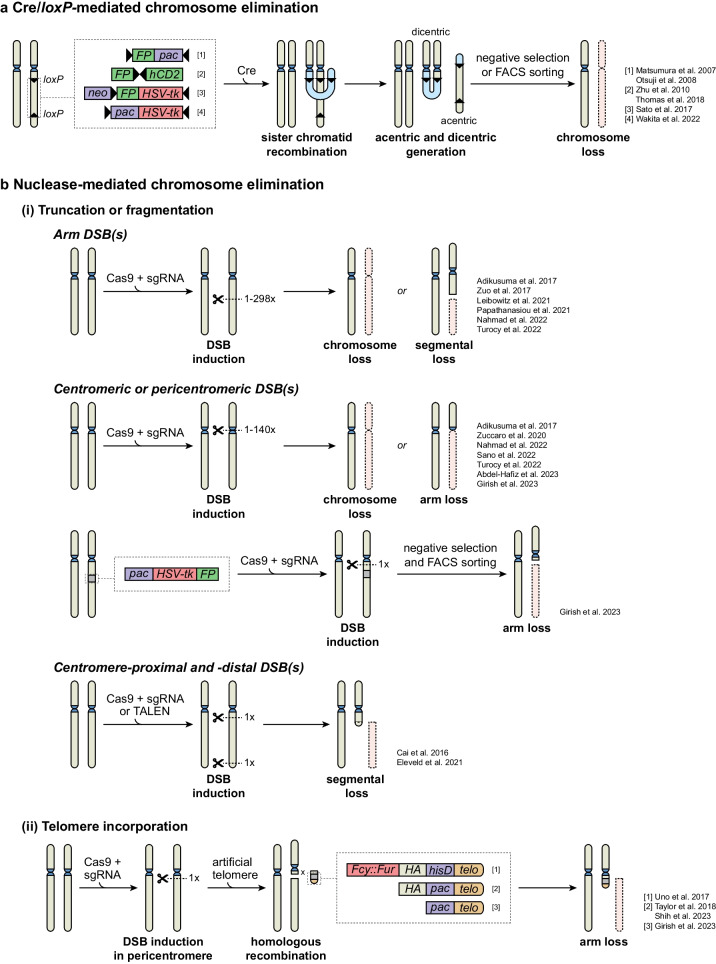
Methods to eliminate specific chromosomes. **a** Two inverted *LoxP* sites are integrated into a chromosome arm of interest. Upon expression of Cre recombinase after S phase, the two sister chromatids can recombine, generating dicentric and acentric chromosomes that are eventually lost after one or multiple mitoses. An antibiotic resistance gene such as pac (puromycin resistance) or neo (neomycin resistance) is usually inserted between the two *loxP* sites to facilitate selection of cells harboring *loxP* integration. To efficiently recover cells with the targeted chromosomal loss, a number of transgenes can be inserted, including ones encoding for fluorescent proteins (FP), cell-surface proteins such as human (h)CD2, or a suicide gene such as herpes simplex virus thymidine kinase (HSV-tk), allowing for FACS sorting or Ganciclovir (GCV)-induced negative selection, respectively. **b** (i) With one or multiple chromosome-specific sgRNAs, one or multiple DNA double stranded breaks (DSBs) are induced either in the arm or in the (peri-)centromere of the targeted chromosome by CRISPR/Cas9, leading to either whole or partial loss of the targeted chromosome. Integration of a suicide gene (i.e., HSV-tk) in the arm of the targeted chromosome can facilitate selection of cells that have lost the targeted chromosome (arm). Alternatively, CRISPR/Cas9 or TALEN can be used to induce two DSBs flanking the chromosomal region to be deleted. This will lead to ligation of the endogenous telomere to the centromere-proximal break site, leading to specific segmental arm loss. (ii) Telomere-mediated chromosome truncation: CRISPR/Cas9 induces a single DSB near the centromere, and the break is repaired using a repair template containing a positive selection marker (pac or L-histidinol dihydrochloride, hisD), a human telomere sequence (telo), and frequently homology arms (HA) overlapping the break site. The incorporation of a synthetic S. *cerevisiae* cytosine deaminase-uracil phosphoribosyl transferase fusion gene (Fcy::Fur) outside the HA, can be used to eliminate cells with an off-target integration by 5-fluorocytosine

Cre recognizes and catalyzes the recombination of *loxP* sites (Sternberg and Hamilton 1981; Sauer and Henderson 1988) and particularly inverted *loxP* sites integrated into a single chromosome (i.e., in cis) give rise to acentric and dicentric chromosome fragments when the *loxP* sites of the duplicated sister chromatids recombine (Lewandoski and Martin 1997). The acentric fragments are lost during successive rounds of cell division following their formation (Ly et al. 2017). The dicentric chromosome fragments, on the other hand, often undergo multiple breakage-fusion-bridge (BFB) cycles before they are lost (Thomas et al. 2018, Fig. 2a). Although not intensively investigated, depending on the chromosome and site of *loxP* integration, these BFB cycles as well as the acentric fragments could in theory also generate oncogene amplifying recombination products, such as extrachromosomal (ec) DNA (Thomas et al. 2018; Warecki and Sullivan 2020; Shoshani et al. 2021b). This potential side-effect needs to be considered when studying the oncogenic potential of Cre/*loxP* generated monosomies (Thomas et al. 2018). Yet, since both the acentric and dicentric chromosome fragments tend to get lost over time, Cre/*loxP* has been widely used to induce targeted whole and partial chromosome loss in vitro and in vivo (Fig. 2a, Supplemental table 3).

*LoxP* sites are frequently co-integrated with a gene encoding a fluorescent protein to facilitate FACS-based enrichment of cells with the desired karyotype (Matsumura et al. 2007; Otsuji et al. 2008; Zhu et al. 2010; Sato et al. 2017; Thomas et al. 2018; Wakita et al. 2022). Alternatively, co-integration of *loxP* with, for instance, the herpes simplex virus thymidine kinase gene (HSV-tk) allows for the negative selection of cells with an integration in the chromosome of interest (Sato et al. 2017; Wakita et al. 2022). Mammalian cells expressing HSV-tk are killed by the anti-viral drug Ganciclovir (GCV) (Borrelli et al. 1988), and thus only cells that have eliminated the chromosome with the integrated HSV-tk gene can survive this treatment. In fact, it even supports the recovery of cells that have spontaneously lost the HSV-tk-bearing chromosome in the absence of Cre-induced recombination, a strategy successfully applied, albeit at very low efficiency, to restore a disomic state of chr21 in Down syndrome induced pluripotent stem cells (iPSCs) (Li et al. 2012). Of note, GCV was recently shown to be mutagenic; thus, cells recovered after GCV selection have likely acquired additional GCV-associated mutations (de Kanter et al. 2021).

Cre/*LoxP* has been extensively used to eliminate stem cell-derived chromosomes from tetraploid hybrids generated by cell fusion of mouse ESCs and differentiated mouse cells. Mouse ESC-somatic cell fusions result in the reprogramming of somatic nuclei to a pluripotent state. By eliminating individual ESC-derived chromosomes from the hybrid, their role in maintaining the pluripotent state was studied (Matsumura et al. 2007; Otsuji et al. 2008). To facilitate this type of stem cell research, a panel of mouse ESC lines with inverted *loxP* sites across 13 different autosomes (mouse chromosomes 1-6, 10-13, 17, 19) and the Y chromosome was generated (Tada et al. 2009). Importantly, Cre/*loxP* was further exploited to generate mice with tissue-specific monosomies via crosses between mice carrying inverted *loxP* sites in a chromosome of interest and mice expressing Cre in a defined cell lineage or tissue. Using this strategy, a copy of chromosome 4, 9, 10, 11, or 14 was eliminated from mouse lymphocytes (Zhu et al. 2010), and a copy of chr2 from the limb buds of mouse embryos (Grégoire and Kmita 2008). Both studies reported cell death in the Cre-expressing tissue, albeit to different extents, likely reflecting chromosome- and tissue-specific responses. Of note, since Cre recombination efficiency varies across tissues partly due to differences in Cre expression levels (Duchon et al. 2008), the induced aneuploidies in mouse tissues are mostly mosaic, meaning that only a fraction of the cells display the intended chromosomal loss (Hérault et al. 2010). Aneuploid mosaicism is observed in both non-transformed and transformed human tissues (Riccardi and Crandall 1978; Hasle et al. 1995; Taylor et al. 2014; Forsberg et al. 2014; Knouse et al. 2014; Fragouli et al. 2017), indicating these mouse models mimic a physiologically relevant condition.

More recently, Cre/*loxP* was used to eliminate one of the three copies of chr21 in HeLa cells, a human hypertriploid cervical cancer line. Interestingly, HeLa cells disomic for chr21 displayed impaired growth compared to the parental trisomic cells (Sato et al. 2017). In marked contrast, Cre/*LoxP*-induced elimination, or transcriptional silencing of the extra copy of chr21 in Down syndrome iPSCs alleviated the proliferative defects linked to trisomy 21 (Li et al. 2012; Jiang et al. 2013). Furthermore, to address the consequences of chromosome loss in the context of cancer, Thomas et al. derived four different tetraploid immortalized MEF cell lines lacking one copy of chromosomes 9, 10, 12, or 14 (Thomas et al. 2018). Except for the line with a loss of chr12, tetraploid MEFs with a chromosome loss displayed enhanced tumorigenic potential compared to isogenic controls. Transformation was associated with ongoing genomic instability in the MEFs with targeted chromosome loss. Importantly, chromosome elimination in diploid cells did not induce transformation, suggesting that a tetraploid background supports tumorigenesis after chromosome loss.

### Chromosome elimination by targetable nucleases

The most simplistic yet versatile way to eliminate a specific chromosome is by inducing multiple DSBs along the arms of a chromosome of interest by CRISPR/Cas9 (Fig. 2b, i). This type of chromosome fragmentation likely produces unrepaired acentric fragments that form micronuclei during cell division and eventually lead to the loss of the targeted chromosome or (segments of) the chromosomal arm (Supplemental table 3) (Leibowitz et al. 2021; Papathanasiou et al. 2021). Multiple chromosome-specific DSBs can be accomplished by one sgRNA targeting chromosome-specific repeats, or by a cocktail of sgRNAs, each targeting a unique sequence. For instance, sex chromosome elimination in mouse ESCs was achieved with relatively high efficiency by directing a single sgRNA towards chromosome-specific repeats in an arm of chrX or chrY (Adikusuma et al. 2017; Zuo et al. 2017). Likewise, chromosome fragmentation effectively achieved loss of chr21 from human Down syndrome iPSCs and of chr7 from the colorectal cancer cell line HT-29 (Zuo et al. 2017).

Alternatively, fragmenting the (peri)centromere seems even more effective (Fig. 2b, i, Supplemental table 3), as inducing multiple breaks in the (peri)centromeric region of chrY induced whole chrY and Y arm loss more efficiently than fragmentation of the long arm of chrY (Adikusuma et al. 2017). In fact, this strategy effectively eliminated mouse chrY from bone marrow cells derived from Cas9 knock-in mice (Sano et al. 2022). When injected into irradiated wild-type recipient mice, the chimeric mice displayed a mosaic loss of chrY in their hematopoietic cells, a condition frequently observed in older males (Forsberg et al. 2014; Dumanski et al. 2016). Strikingly, these mice displayed shorter life spans and developed age-associated cardiomyopathies (incl. myocardial fibrosis) earlier in life than control mice, attributed to chrY-deficient macrophages in the heart that somehow overactivated a pro-fibrotic signaling network (Sano et al. 2022).

While the identification of centromere-specific sgRNAs was a challenge in the past, the recent publication of the T2T genome assembly now enables researchers to excavate targetable centromeric repeats, thereby expanding the range of chromosomes that can be eliminated with this strategy (Altemose et al. 2022; Nurk et al. 2022; Bosco et al. 2023). Furthermore, inducing two DSBs flanking the targeted chromosome arm (one DSB proximal and one distal to the centromere) by CRISPR/Cas9 or TALEN was shown to efficiently delete large parts of chromosomal arms, most likely because the distal chromosome fragment harboring the telomere recombined and capped the truncated chromosome (Fig 2b, i). This approach was used to eliminate segments of chr8p from non-malignant MCF10A mammary epithelial cells (Cai et al. 2016), and of chr11q and chr6q from human neuroblastoma SKNSH and NMB cells (Eleveld et al. 2021, Supplemental table 2).

Finally, several recent reports detected specific chromosome loss as an on-target, undesirable side-effect of a single DSB induced by CRISPR/Cas9-mediated gene editing (Zuccaro et al. 2020; Leibowitz et al. 2021; Papathanasiou et al. 2021; Turocy et al. 2022; Nahmad et al. 2022, Supplemental table 3). In fact, CRISPR/Cas9 targeting of single genes caused both whole-chromosome and segmental loss of chromosomes 2, 5, 6, and X in hTERT-RPE1 cells (Leibowitz et al. 2021); chr2 and chr17 in mouse embryos (Papathanasiou et al. 2021); segmental loss of chr7 and either gain or loss of whole chr14 in human primary T cells (Nahmad et al. 2022). Specific losses were detected at low frequencies shortly after the first cell division cycles following CRISPR/Cas9 and sgRNA expression. Interestingly, a relatively high incidence (over 35%) of whole or partial losses of chromosomes 6, 16, 17, or X could be achieved in human embryos after inducing a single DSB near the centromere of these chromosomes in either pre-fertilized oocytes or 2-cell stage zygotes (Zuccaro et al. 2020; Turocy et al. 2022). Thus, targeted single DSBs, especially the ones near or in the centromere, can be leveraged to either generate specific monosomies, or correct specific trisomies in mammalian cancerous and developmental models. It is important to realize, however, that additional rearrangements involving the targeted chromosome take place or precede the chromosomal loss (Leibowitz et al. 2021; Papathanasiou et al. 2021; Turocy et al. 2022). Moreover, how well recently generated monosomies can be stably maintained depends on the cell type and genetic background in which these losses are generated. Both human and mouse embryos seem to exhibit greater tolerance for monosomies compared to cultured ESCs, as evidenced by the detection of both arm-level and whole-chromosome losses in trophectoderm biopsies of blastomeres, and the unsuccessful attempts to derive monosomic ESCs from these early embryos (Biancotti et al. 2012; Zuccaro et al. 2020). Furthermore, the loss or inactivation of TP53 is an important factor to recover somatic human cell lines with stable monosomies (Chunduri et al. 2021).

An advantage of the CRISPR/Cas9 based approaches is that they do not require prior engineering of the chromosome of interest. This makes them in principle versatile and translatable to many different cell types, provided that these cells can be efficiently transfected or transduced with CRISPR/Cas9 and expanded from single cell cultures. However, this is often a problem and hence complementary strategies allowing for enrichment of cells with the engineered loss have been developed, such as telomere-mediated arm truncation (Fig. 2b, ii). Here, a centromere-proximal DSB is induced, and cells are provided with a repair template harboring an artificial telomere (Uno et al. 2017; Taylor et al. 2018). This repair template contains 100–1000 bp of human telomeric seed sequence, a homologous sequence to the targeted region, and a positive selection marker (often a puromycin resistance gene) to facilitate the recovery of cells with neo-telomere incorporation. Additionally, a negative selection marker incorporated into the repair template outside the homology arm can be used to eliminate cells with random integration of the artificial telomere (Uno et al. 2017). Using such an approach, Taylor et al. deleted chr3p from squamous lung cancer cells to model its recurrent loss in this cancer type and found that the mere loss of chr3p was not sufficient to induce transformation (Taylor et al. 2018). In addition, this strategy was used to delete chr8p from immortalized lung epithelial cells to validate the BISCUT algorithm prediction that non-homozygous loss of the DNA helicase WRN contributes to the positive selection of chr8p loss in cancer (Shih et al. 2023). Alternatively, a negative selection marker such as HSV-tk can be integrated into the chromosome (arm) of interest prior to induction of a peri-centromeric DSB (Fig. 2b, i). Although this requires additional chromosome engineering, it allows for selection of cells that have lost the chromosome (arm) of interest, for example by GCV treatment (discussed above). This strategy allowed Girish et al. to correct trisomy 1q in several cancer cell lines including A2058 (melanoma), AGS (gastric cancer), and A2780 (ovarian cancer). Restoring the chr1q disomic state in these cell lines reduced their anchorage-independent growth ability in vitro and in vivo (Girish et al. 2023). This was partly attributed to over-expression of the TP53 inhibitor MDM4 and the Wnt/β-catenin effector BCL9 in trisomic 1q cells, making these cells addicted to this specific aneuploidy in an oncogene-like manner. Moreover, the isogenic trisomic-disomic 1q cell lines provided a platform to investigate potential therapeutic vulnerabilities of cells with a chr1q gain. Over-expression of the pyrimidine salvage kinase UCK2 caused by the 1q trisomy, rendered these cells especially sensitive to the nucleotide analogs RX-3117 and 3-deazauridine compared to their disomic counterparts (Girish et al. 2023).

## Inducing, detecting, and isolating cells with specific chromosomal gains *and* losses

**Fig. 3:**
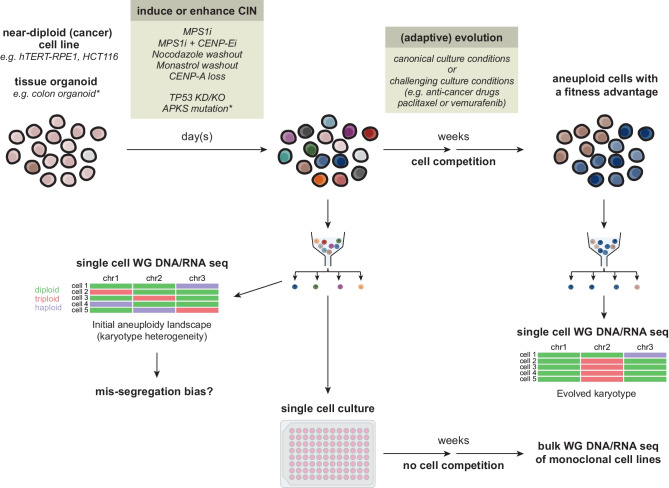
Approaches to induce, detect, and isolate cells with specific aneuploidies. Non-transformed or transformed near-diploid cells with or without functional TP53 can acquire heterogeneous aneuploid karyotypes after transient or chronic induction of CIN by compounds that disrupt the chromosome segregation machinery. In addition, the mere knockout (KO) or knockdown (KD) of TP53 in hTERT-RPE1 cells or sequential mutation of APC, TP53, KRAS and SMAD4 (APKS) in colorectal organoids is sufficient to increase CIN. FACS-based single-cell sorting followed by WG DNA or RNA sequencing shortly after CIN induction permits assessment of karyotype heterogeneity in the initial aneuploidy landscape thereby revealing potential mis-segregation biases. At the same time, single cell culture after CIN induction can generate monoclonal lines harboring specific monosomies or trisomies. These can be subjected to bulk WG DNA or RNA sequencing to reveal their (altered) karyotypes. Finally, the initially heterogeneous and mosaic aneuploid population can be further cultured under standard conditions or under certain challenging conditions such as anti-cancer drugs. Aneuploidy patterns that eventually emerge can be detected by single-cell WG DNA or RNA sequencing. Of note, WG RNA sequencing, carried out either shortly after CIN induction or after prolonged culture, also allows for analyses of cellular responses to specific chromosomal gains and losses. i, inhibitor. This figure was partially created with Biorender

Most of the strategies described above require (prior) genetic engineering of the chromosome of interest, and/or extensive clonal expansion of cells with the intended karyotype change. Alternative approaches involve the isolation and analysis of cells after experimentally inducing chromosome segregation errors (CIN, Fig. 3). In cultured mammalian cells, CIN is most frequently induced or enhanced by disturbing the chromosome segregation machinery. This disruption can be achieved by exposing cells to compounds that interfere with microtubule dynamics (e.g., paclitaxel, nocodazole, and Aurora B kinase inhibitors), prevent the formation of a bipolar mitotic spindle (e.g., Eg5 inhibitors like monastrol) or impede the mitotic checkpoint, such as MPS1 kinase inhibitors (Mayer et al. 1999; Cimini et al. 2001; Ditchfield et al. 2003; Hauf et al. 2003; Lampson et al. 2004; Santaguida et al. 2010; Weaver 2014; Maia et al. 2018). The mitotic checkpoint ensures that anaphase only begins after kinetochores (i.e., multi protein structures that assemble on centromeres and function as microtubule binding sites of the chromosomes) have properly attached to the mitotic spindle (Musacchio and Salmon 2007). Checkpoint inactivation is sometimes combined with inhibition of the kinetochore-localized kinesin CENP-E, to diminish chromosome congression and favor whole chromosome mis-segregations (Weaver et al. 2003; Soto et al. 2017). Additionally, several CIN-induced aneuploidy mouse models have been generated through overexpression, heterozygous deletion, or mutation of mitotic checkpoint proteins, or by overexpression of Polo-like-kinase 4 (PLK4) to induce centrosome amplification (Michel et al. 2001; Sotillo et al. 2007; Weaver et al. 2007; Iwanaga et al. 2007; Li et al. 2009; Baker et al. 2009; Foijer et al. 2014; Levine et al. 2017; Hoevenaar et al. 2020). Collectively, these and other in vivo models demonstrated that depending on the level of CIN, tissue context and genetic background, CIN can either suppress or promote carcinogenesis (Schvartzman et al. 2010; Simon et al. 2015). More recently, some of these mouse models were refined to more tightly control the level and duration of CIN in adult mouse tissues (Foijer et al. 2017; Trakala et al. 2021; Shoshani et al. 2021a). Only these latest mouse models are discussed below.

Evidently, these in vitro and in vivo CIN-inducing strategies do not permit precise control over the identity of the chromosome that mis-segregates. However, they generate a population of cells with heterogenous aneuploid karyotypes. Via single-cell whole genome (WG) DNA or RNA sequencing (Bakker et al. 2016; Zhao et al. 2019; Kashima et al. 2020), or by chromosome fluorescence in situ hybridization (FISH) in combination with image-based flow cytometry (Image-Stream, Worrall et al. 2018), the aneuploidy landscapes and cellular responses have been analyzed shortly after the mis-segregation events or after a period of adaptation in either standard or challenging in vitro culture conditions, or in vivo environments (Fig. 3).

### Conditions resulting in chromosome mis-segregation biases

Single-cell whole genome DNA sequencing (scWGS) and high-throughput chromosome FISH applied to cultured human cells shortly after CIN induction showed that certain chromosomes have a higher tendency to mis-segregate than others. In fact, it supported earlier conclusions based on FISH with a limited set of chromosome-specific probes that distinct chromosome mis-segregation biases are seen depending on cell type and the mode of CIN induction (Drpic et al. 2018; reviewed in Klaasen and Kops 2022). For example, temporary depolymerization of microtubules (MT) by nocodazole treatment and washout elevated the mis-segregation rates of chr1 and 2 more than that of other chromosomes in non-transformed hTERT-RPE1 and BJ-hTERT cells (Worrall et al. 2018). These large chromosomes appeared to be prone to cohesion fatigue, the gradual failure to maintain sister-centromere cohesion during a mitotic delay (Daum et al. 2011), in this set-up caused by the nocodazole-induced spindle disruption and consequent activation of the mitotic checkpoint (Worrall et al. 2018).

Second, inducible degradation of the centromere-specific histone CENP-A was shown to specifically perturb kinetochore function and faithful segregation of the human Y chromosome in the male DLD1 colorectal cancer cell line. This is because the human chromosome Y centromere harbors alpha satellite DNA repeats that cannot bind CENP-B, and CENP-B temporarily maintains kinetochores in the absence of CENP-A (Fachinetti et al. 2015). This unique property of the Y chromosome centromere may account for its slightly higher mis-segregation rate compared to chromosome X or 4, even in the presence of CENP-A (Fachinetti et al. 2015). Furthermore, it may contribute to the observed mosaic loss of chrY in peripheral blood lymphocytes of ~20% of the male population (Thompson et al. 2019; Lau 2020), and to the frequent loss of chrY in a variety of tumor types in males (Qi et al. 2023). Interestingly, centromeres of the other chromosomes vary in their number of CENP-B binding sites (Earnshaw et al. 1989; Dumont et al. 2020). In the absence of CENP-A, this is reflected in differences in “kinetochore strengths” per chromosome. Accordingly, large chromosomes with the least CENP-B binding sites and hence weakest kinetochores (i.e., chr3, 6, and X) were found to mis-segregate more often in female hTERT-RPE1 cells lacking CENP-A. Conversely, small chromosomes with large centromeres (i.e., chr 17–20) mis-segregated the least under these conditions (Dumont et al. 2020). Whether these mis-segregation biases caused an equal increase in both losses *and* gains of these specific chromosomes in the population is currently not clear.

Finally, by combining scWGS with individual chromosome tracking and manipulation, Klaasen et al. showed that human chromosomes occupying the periphery of the interphase nucleus have a higher probability to mis-segregate in hTERT-RPE1 cells when MPS1 is inhibited (Klaasen et al. 2022). These include the larger chromosomes (1–5), but also the smaller chr18. Peripheral chromosomes are thought to more frequently end up near or behind the spindle poles at the beginning of mitosis, and therefore take longer to congress and bi-orient on the mitotic spindle (Klaasen et al. 2022). In line with this, larger chromosomes were also more often stalled near the spindle poles after inhibition of CENP-E, the MT plus-end directed kinesin that facilitates chromosome transport from the spindle pole to the equator (Kapoor et al. 2006; Tovini and McClelland 2019).

It is currently not clear whether these chromosome mis-segregation biases contribute to the establishment of cancer-associated aneuploidy patterns. Since mis-segregating chromosomes can also form micronuclei that can trigger chromothripsis (Stephens et al. 2011; Crasta et al. 2012; Zhang et al. 2015; Ly et al. 2017; Umbreit et al. 2020), chromosomes that mis-segregate more frequently than others may instead be over-represented in chromothriptic recombinations observed in certain cancers (Cortés-Ciriano et al. 2020; Klaasen et al. 2022).

### Specific aneuploidies evolving after CIN induction

As mentioned, the (transient) induction of CIN generates a population of cells with a variety of aneuploid karyotypes that can serve as substrates for (adaptive) evolution. WGS of a significant number of single cells sampled from the population at various time points during adaptation will subsequently reveal the number and type of aneuploidies that evolve and dominate the culture during adaptation and selection (Fig. 3). Because cells with and without aneuploid genomes co-exist in the initial population, the dominant aneuploidies are most likely selected because they confer a fitness advantage. Such an approach demonstrated that segmental aneuploidies, a consequence of chromosome breakage, are almost exclusively tolerated and propagated in cells that lack functional TP53 (Santaguida et al. 2017; Soto et al. 2017). Moreover, it uncovered that acquired resistance to a variety of chemotherapeutic drugs is associated with the selection of cells harboring specific chromosome gains and/or losses (Ippolito et al. 2021; Lukow et al. 2021). For example, resistance to paclitaxel of hTERT-RPE1 cells correlated with a stable gain of chr11 or loss of chr10 (Ippolito et al. 2021; Lukow et al. 2021). In contrast, resistance to the BRAF inhibitor vemurafenib recurrently selected for a gain of chr7 in the BRAF mutant colorectal cancer cell line Colo205, but for recurrent gains of chr11 and 18 in A375, a BRAF mutant melanoma cell line (Lukow et al. 2021).

Of note, the mutation or knock-down of TP53 in the hTERT-RPE1 cell line supports clonal outgrowth of cells with specific karyotypes, either with or without experimentally increasing chromosome mis-segregation rates (Chunduri et al. 2021; Hintzen et al. 2022; Adell et al. 2023) (Fig. 3). Monoclonal cell lines with a single monosomy for chr10, 13, 19p, X, or double monosomies for chr10;18 or chr7;10 could be derived after single-cell culture of hTERT-RPE1 cells in which TP53 was inactivated, albeit at low frequency. Re-expression of TP53 in these cell lines suppressed their viability and proliferative capacity, suggesting that TP53 acts as an important barrier against the proliferation of cells with chromosome loss (Chunduri et al. 2021). Similarly, sequential mutation of APC, TP53, KRAS and SMAD4 (APKS) by CRISPR/Cas9 in colorectal organoids derived from healthy human tissues also supported the evolution of specific aneuploidies. Particularly, aneuploidies frequently observed in colorectal cancers, such as monosomies of chr18, 8p, and 4, emerged and dominated after long-term culture of the APKS organoids (Kester et al. 2022).

Furthermore, in adult mouse tissues, heterogeneous and mosaic aneuploidies were observed three months after CIN induction by bypassing the mitotic checkpoint via inducible expression of a mutant form of the APC/C activator CDC20 that cannot bind MAD2 (CDC20AAA) (Trakala et al. 2021). However, the T cell lymphomas that developed in these animals later in life displayed characteristic and recurrent gains of chr14 and 15, together with less frequent gains and losses of other chromosomes (Trakala et al. 2021). Importantly, the frequent gain of chr15 was attributed to the presence of c-MYC on this chromosome, and expressing this oncogene from chr6 was sufficient to select for trisomy of chr6 instead of chr15 (Trakala et al. 2021). Similarly, T cell lymphomas that developed in TP53+/− or TP53−/− mice also exhibited recurrent gains of chr15, yet often accompanied by gains of chr4, 5, and 14. These aneuploidies arose after either transient induction of CIN via temporary PLK4 overexpression (Shoshani et al. 2021a), or after chronic CIN induced in the T cell compartment (Foijer et al. 2017; Shoshani et al. 2021a). Collectively, these examples show that under certain in vitro and in vivo selection pressures, CIN-induced heterogeneous and mosaic aneuploidies can evolve towards more homogeneous, cell-type specific aneuploidies.

Taken together, scWGS and bulk WGS of individual cell clones are powerful technologies to assess the karyotype of cell populations or monoclonal cell lines, respectively. By revealing the level of aneuploidy per individual chromosome and the level of aneuploid mosaicism in a cell population following various CIN-inducing treatments in different cell lines (Bakker et al. 2016), these analyses provide a starting point for understanding how cancer type-specific aneuploidy patterns arise and evolve, and how specific aneuploidies may contribute to drug resistance and tumor formation. Importantly, ongoing developments in computational methods to analyze single-cell sequencing data have made it possible to reliably deduce chromosome copy number states from single-cell RNA sequencing data (Patel et al. 2014; Bosco et al. 2023). This development enables the linking of cellular states and responses to specific chromosomal gains and losses in cell lines, organoids, and cancer tissues (De Falco et al. 2023; Gao et al. 2023).

*dCas9-based approaches to mis-segregate specific human chromosomes*

**Fig. 4:**
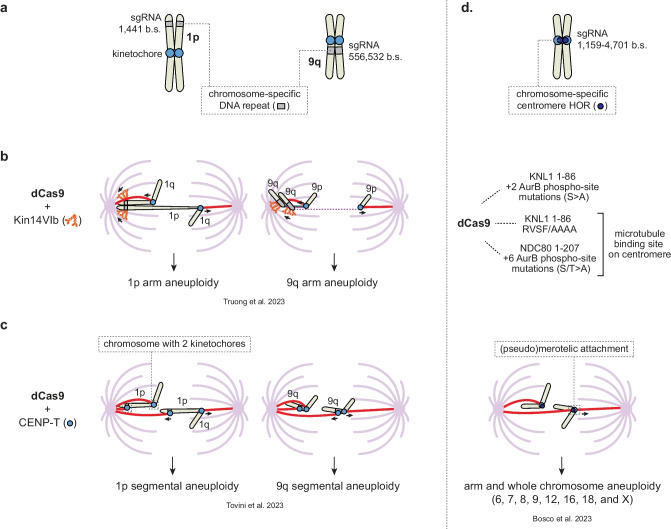
dCas9-based methods for mis-segregating specific human chromosomes. **a**–**c** dCas9 fused to either P. *patens* Kinesin14VIb (Kin14VIb) (**b**), or human CENP-T^1-243^ (**c**) has been used in combination with a sgRNA targeting chromosome-specific DNA repeats in chr1 and 9 for inducing chromosome-specific mis-segregation and aneuploidy. **d** A third strategy targets chromosome-specific higher order repeats (HOR) of centromeres with unique sgRNAs to recruit dCas9 fused to either KNL1^1-86/S24A;S60^, KNL1^1-86/RVSF/AAAA^, or NDC80^1-207/6xS/T>A^. We propose that dCas9-KNL1^-86/RVSF/AAAA^ and dCas9-NDC80^1-207/6xST>A^ together with a centromere-specific sgRNA create an ectopic microtubule binding site on the centromere, which can lead to (pseudo-) merotelic attachment of the targeted chromosome during mitosis. Whole chromosome and chromosomal arm aneuploidies for chr6, 7, 8, 9, 12, 16, 18, and X could be generated using this approach. Number of predicted sgRNA binding sites (b.s.) based on the T2T genome assembly are indicated in a, d. AurB, Aurora B

Instead of inducing multiple, mostly random chromosome mis-segregations per division and retrospectively assessing the identity of the aneuploid chromosomes in the progeny, very recent approaches have attempted to prospectively mis-segregate a single specific chromosome for inducing its respective gain and loss in the daughter cells (Bosco et al. 2023; Tovini et al. 2023; Truong et al. 2023). The common principle of these approaches is the use of a nuclease-dead Cas9 (dCas9) and a sgRNA to tether a certain protein to a chromosome of interest. This protein then interferes with the faithful segregation of that chromosome during cell division (Fig. 4a–d). dCas9 is directed by the sgRNA to a chromosome-specific repetitive DNA sequence, repeats that are present in both homologs and predominantly found near the telomere, the pericentromere, or underlying the centromere (Bosco et al. 2023; Tovini et al. 2023). As the complementary sgRNA sequence is sometimes present in the repeat over 1000 times, many dCas9 molecules can accumulate on the repeat using one unique sgRNA. Thus far, the following strategies have been tested.

#### Counteracting chromosome congression

Through tethering of a MT minus-end directed motor protein onto a chromosome of interest, Truong and Cané-Gasull et al. aimed to counteract the forces that drive chromosome congression and to cause a selected chromosome to misalign and mis-segregate during metaphase and anaphase, respectively (Truong et al. 2023, Fig. 4b). The motor and stalk domain of Kinesin 14VIb (Kin14VIb) from the land moss Physcomitrella *patens* (Jonsson et al. 2015; Nijenhuis et al. 2020) were indirectly bound to dCas9 via rapalog-induced FRB-FKBP12 heterodimerization. dCas9 and kinesin were directed by specific sgRNAs to either a subtelomeric repeat of chr1p or a large pericentromeric repeat of chr9q in hTERT-RPE1 cells with functional TP53. Despite poleward transport of the Kin14VIb-bound locus during (pro)metaphase, the kinetochores of the kinesin-bound sister chromatids acquired bi-oriented MT attachments that silenced the mitotic checkpoint. The counteracting pulling forces caused by Kin14VIb motors walking towards one spindle pole and kinetochore MTs attached to the opposite spindle pole caused typical morphological changes of the targeted chromosomes. In the case of chr1, the 1p arm was heavily stretched in anaphase, while in the case of chr9, the Kin14VIb-bound pericentromere and 9q arm became separated from the kinetochore and 9p arm during metaphase and anaphase (Fig. 4b). This eventually led to arm-level aneuploidies of either 1p or 9q after a single cell division (Truong et al. 2023). While the 9q aneuploidies included tetrasomies, nullisomies, and monosomies, the number of cells with trisomies was relatively low, for reasons not yet well understood.

#### Ectopic kinetochore assembly

Inspired by prior LacO-LacI tethering studies demonstrating that the N-terminus of Centromere Protein-T (CENP-T^1-243^) is sufficient to assemble a functional kinetochore on an integrated LacO repeat (Gascoigne et al. 2011; Gascoigne and Cheeseman 2013), Tovini et al. fused CENP-T^1-243^ to dCas9 to create an extra kinetochore on a chromosome of interest (Fig. 4c, Tovini et al. 2023). Indeed, transfection of dCas9-CENP-T in HEK293T and HCT116 cells together with the same sgRNAs for chr1 and chr9 as described above recruited high levels of the kinetochore proteins NDC80/HEC1 and KNL1 near the telomere of chr1p and to the pericentromere of chr9q, respectively. These ectopic kinetochores attached to spindle MTs and activated the mitotic checkpoint, and MPS1 was therefore inhibited to promote anaphase onset and the mis-segregation of the targeted chromosome. Single-cell sequencing of HEK293T cells shortly after expressing dCas9-CENP-T revealed an increase in a range of large copy number alterations for chr1p and chr9q, compared to cells expressing dCas9 that was not fused to CENP-T (Tovini et al. 2023). Thus, assembly of an extra kinetochore either close to the telomere or nearby the native kinetochore generates segmental aneuploidies of the targeted chromosome.

#### Centromere targeting

Bosco et al. developed an elaborate computational pipeline to analyze the T2T human genome assembly and identify sgRNAs targeting chromosome-specific alpha-satellite centromeric repeats (Altemose et al. 2022; Nurk et al. 2022; Bosco et al. 2023). This analysis delivered sgRNAs targeting the centromeres of 15 different chromosomes (2, 4, 5-13, 16, 18, 19, X). sgRNAs selected based on their ability to recruit dCas9 at centromeres by imaging was used to dock either the N-terminal MT binding domain (aa 1-207) of NDC80/HEC1 (DeLuca et al. 2006), or the N-terminus (aa 1-86) of KNL1 onto centromeres via direct fusion to dCas9 (Bosco et al. 2023) (Fig. 4d). While all dCas9 fusion proteins appeared effective, dCas9-KNL1^1-86/RVSF/AAAA^ was studied most extensively. Its expression in hTERT-RPE1-CDKN1A/RB1 knock-down or hCEC-TP53KO cells together with sgRNAs for chr7 or chr18 induced the mis-segregation of these chromosomes during mitosis and their aneuploidies in a significant fraction of the cells. Impressively, with their strategy, Bosco et al. successfully generated not only segmental, but also whole-chromosome gains and losses of various other specific chromosomes (chr6, 8, 9, 12, 16, and X)(Bosco et al. 2023). Although successful, how centromere-docking of dCas9-KNL^1-86/RVSF/AAAA^ induces chromosome mis-segregation remains unclear. KNL1^1-86^ can bind to MTs and to protein phosphatase 1 (PP1) via its SSILK and RVSF motifs (Liu et al. 2010; Bajaj et al. 2018). PP1 recruitment by KNL1 supports mitotic checkpoint silencing and presumably the stabilization of kinetochore MT attachments by dephosphorylating MPS1 and Aurora B substrates within the kinetochore (Liu et al. 2010; Zhang et al. 2014; Nijenhuis et al. 2014). Phosphorylation of the SSILK and RVSF motifs of KNL1 by Aurora B, on the other hand, counteracts PP1 recruitment, thereby reinforcing mitotic checkpoint signaling and potentially kinetochore MT destabilization in early mitosis (Liu et al. 2010; Nijenhuis et al. 2014; Nasa et al. 2018). Bosco et al. propose that recruiting a small N-terminal KNL1 fragment with a mutated RVSF motif to centromeres disrupts the Aurora B:PP1 balance, such that Aurora B decreases the stability of kinetochore MT attachments on the chromosome of interest (Bosco et al. 2023). It remains, however, difficult to envision how a small part of KNL1, unable to bind PP1, and tethered to the centromere, can interfere with endogenous KNL1-PP1 at the kinetochore. Since KNL1^1-86^ also binds MTs, especially in the absence of PP1 (Espeut et al. 2012; Bajaj et al. 2018), a more likely scenario may be that dCas9-KNL^1-86/RVSF/AAAA^, in combination with centromere-specific sgRNAs, creates an ectopic MT binding site on the centromere similar to dCas9-NDC80^1-207^. This extra MT binding site on the centromere may increase the risk of acquiring a (pseudo) merotelic attachment (i.e., a single chromatid bound by MTs emanating from opposing spindle poles), that causes the targeted chromosome to lag and mis-segregate during anaphase. Alternatively, dCas9 binding is known to act as a roadblock during replication, especially on repetitive DNA sequences (Whinn et al. 2019; Doi et al. 2021), and it might as well be that the mere binding of dCas9-KNL1^1-86/RVSF/AAAA^ causes replication or transcriptional problems that result in incomplete replication or inactivation of the centromere. Irrespective of the mechanism, dCas9-based targeting of centromeric repeats appears to work very efficiently to induce both whole and partial specific chromosomal gains and losses (Bosco et al. 2023).

The advantages of the dCas9-based approaches are that they do not require prior engineering of the chromosome of interest, are applicable to many different cell types, and in principle can generate segmental as well as whole chromosome gains and losses, depending on the chromosomal region targeted. The main challenges lie in expressing sufficient levels of the dCas9-fusion proteins and in identifying sgRNAs that are not only chromosome-specific, but also able to bind to a chromosomal region at least 1000 times. Either way, this dCas9-based toolbox opens up new avenues to manipulate individual mitotic chromosomes and to systematically test the immediate and late cellular responses of various tissue types to the gain and loss of a single specific chromosome.

## Conclusion and future perspectives

In this review, we aimed to provide a complete and updated overview of the current methods and technologies for inducing specific aneuploidies in mouse and human cell systems. The past decade has witnessed an explosion of novel and improved strategies for manipulating human and mouse chromosomes and karyotypes. With the development of various CRISPR/Cas9 and dCas9-based approaches, as well as state-of-the-art WG DNA and RNA-sequencing methods (i.e. single-cell and spatial transcriptomics, Erickson et al. 2022), a large toolbox is now available to generate, isolate, detect, and study specific aneuploidies in healthy and diseased mammalian tissues. Moreover, for many of the described approaches (MMCT, CENP-A loss, CRISPR/Cas9, and dCas9-based methods), chromosome gain or loss is accompanied by micronucleus formation of the targeted chromosome. Hence, these methods may also provide unique opportunities to investigate the faith of micronuclei with known chromosome content (Ly et al. 2017; Ly et al. 2019; Kneissig et al. 2019; Leibowitz et al. 2021; Papathanasiou et al. 2021; Bosco et al. 2023; Truong et al. 2023). In fact, recent findings suggests that the content of a micronucleus can determine if and when it will rupture in the following cell cycle (Mammel et al. 2022). Additionally, chromothripsis of specific micronucleated chromosomes can drive oncogenic amplification and drug resistance in cancer through ecDNA generation (Shoshani et al. 2021b).

These methods also offer the possibility to address whether certain tissues tolerate particular chromosomal gains or losses better than others by engineering a specific aneuploidy in a mouse tissue of interest via inducible and tissue-specific expression of either Cre recombinase, active Cas9, or nuclease-dead Cas9. Additionally, transplantation of mouse cancer cells with an engineered karyotype into isogenic immuno-competent mice allows assessment of how specific aneuploidies affect metastasis formation or remodel the tumor microenvironment. In fact, injection of a mouse bladder cancer cell line with a CRISPR/Cas9-engineered loss of chrY, into immunocompetent male C57BL/6 mice, revealed that tumors lacking chrY are more efficient in evading anti-tumor adaptive immunity because these tumors promote the dysfunction of CD8+ T cells in their tumor microenvironment (Abdel-Hafiz et al. 2023). Finally, introducing these chromosome manipulation methods into gastruloids or other embryonic models (Shahbazi et al. 2019; van den Brink and van Oudenaarden 2021; Oldak et al. 2023; Weatherbee et al. 2023) will enable studies on how (stem) cell fate decisions are affected by certain karyotype alterations during mouse and human development.

While the generation of models for specific aneuploidies forms an essential step to evaluate their impact on developmental and cancer biology, additional analyses and manipulations are required to pinpoint the underlying mechanisms by which specific chromosomal gains or losses contribute to cancer in particular tissue or genetic contexts. By analyzing differentially expressed genes in response to specific aneuploidies, researchers uncovered a link between chr1q gain and MDM4-mediated TP53 suppression in cancer cells (Girish et al. 2023) and validated that chr18q loss can drive TGF-β resistance in colon cancer (Bosco et al. 2023). Elaborate computational algorithms can be applied to identify candidate genes underlying the phenotypes driven by specific aneuploidies. For instance, the TUSON Explorer algorithm, which predicts cancer drivers based on their mutational patterns, can be combined with copy number analysis to identify potential driver genes on recurrently gained or lost chromosomes in cancer (Davoli et al. 2013). In addition, weighted correlation network analysis (aka WGCNA) can be used to identify overexpressing genes that correlate with a specific chromosomal gain (Su et al. 2021). Candidate genes can subsequently be validated by RNAi or CRISPRa/CRISPRi screens using libraries of shRNA, siRNAs, or sgRNAs targeting coding genes on the chromosome of interest (Xue et al. 2012; Gilbert et al. 2013; Gilbert et al. 2014; Bock et al. 2022), or by transduction of a library of bar-coded open reading frames (ORF) (Sack et al. 2018; Su et al. 2021). Through a combination of these approaches, Su et al. demonstrated that dose-sensitive overexpression of RAD21 caused by trisomy 8 helped mitigate the replication stress induced by the oncogenic EWS-FLI1 fusion in Ewing sarcoma (Su et al. 2021). Next to this, integration of XIST, the long non-coding RNA (lncRNA) that inactivates one of the X chromosomes in females (Boumil and Lee 2001; Engreitz et al. 2013; Simon et al. 2013), was found to fully inactivate a copy of chr21 (Jiang et al. 2013; Chiang et al. 2018; Czermiński and Lawrence 2020), and to silence parts of mouse chr1 and human chromosomes 1p, 3q, 4q, 7p, 7q, 8p, 12q, and 15q (Kelsey et al. 2015; Loda et al. 2017; Naciri et al. 2021). Although less specific than RNAi or CRISPRi, the epigenetic silencing potential of this lncRNA could be leveraged to pinpoint which part of the chromosome of interest is responsible for certain aneuploidy-related phenotypes.

With the current possibilities to generate customized karyotypes in various cell types in a dish or whole organism, exciting times lie ahead for the field. Together with functional genetic screens and elaborate computational pipelines to analyze large WG DNA and RNA-sequencing data sets of patient-derived cancer tissues it will fuel advancements in understanding how cancer cells tolerate and benefit from aneuploidy, and how specific aneuploidies impact development.

### Supplementary information

ESM 1 (PDF 139 kb)

## Data Availability

Data sharing is not applicable to this article as no datasets were generated or analyzed during the current study.

## Abbreviations

APC: adenomatous polyposis coli
APC/C: anaphase promoting complex/cyclosome
APKS: APC, TP53, KRAS and SMAD4
AurB: Aurora B
BCL9: B-cell lymphoma 9
BFB: breakage-fusion-bridge
BISCUT: breakpoint identification of significant cancer undiscovered targets
BJ-hTERT: hTERT immortalized human BJ foreskin fibroblasts
BRAF: rapidly accelerated fibrosarcoma homolog B
CD: cluster of differentiation
CDC20: cell division cycle protein 20
CDKN1A: cyclin dependent kinase inhibitor 1A
hCEC: human colonic epithelial cells
CHO: Chinese hamster ovary
CENP: centromere protein
chr: chromosome
CIN: chromosomal instability
c-MYC: cellular myelocytomatosis oncogene
Cre: causes/cyclization recombination
CRISPR: clustered regularly interspaced short palindromic repeats
CRISPRa: CRISPR activator
CRISPRi: CRISPR interference
dCas9: nuclease-dead Cas9
Dox: doxycyclin
DSB: DNA double-stranded break
ecDNA: extra-chromosomal DNA
ESC: embryonic stem cell
EWS: Ewing sarcoma
FACS: fluorescence activated cell sorting
Fcy::Fur: S. cerevisiae cytosine deaminase-uracil phosphoribosyl transferase fusion gene
FISH: fluorescence in situ hybridization
FKBP12: FK506 binding protein 12
FLI: friend leukemia virus integration 1
FP: fluorescent protein
FRB: FKBP12–rapamycin-binding
GCV: ganciclovir
HA: homology arm
HEC1: highly expressed in cancer 1; mammalian homolog of NDC80
HEK293T: human embryonic kidney 293T
HisD: L-histidinol dihydrochloride
HSV-tk: herpes symplex virus thymidine kinase
hTERT: human telomerase reverse transcriptase
hTERT-RPE1: hTERT immortalized retinal pigment epithelial cell line
iPSC: induced pluripotent stem cell
KD: knockdown
Kin14VIb: physcomitrella patens kinesin14VIb
KNL1: kinetochore scaffold 1
KO: knockout
KRAS: Kirsten rat sarcoma viral oncogene homolog
LacI: lac inhibitor
LacO: lac operon
lncRNA: long non-coding RNA
LOH: loss of heterozygosity
loxP: locus of crossing (x) over, P1
MAD2: mitotic arrest deficient 2
MDM4: murine double minute 4
MEF: mouse embryonic fibroblast
MMCT: microcell-mediated chromosome transfer
M.m. domesticus: mus musculus domesticus
MPS1: monopolar spindle 1
MT: microtubule
neo: neomycin resistance gene
NDC80: nuclear division cycle 80
ORF: open reading frame
pac: puromycin resistance gene (puromycin N-acetyltransferase)
PLK4: polo-like kinase 4
PP1: protein phosphatase 1
RB1: retinoblastoma protein 1
Rob: Robertsonian chromosome
rtTA: reverse-tet transactivator
Seq: sequencing
sgRNA: single guide RNA
shRNA: small hairpin RNA
siRNA: small interference RNA
SMAD4: mothers against decapentaplegic homolog 4
SPG20: spastic paraplegia 20
T2T: telomere to telomere
TALEN: transcription activator-like effector nuclease
Telo: telomere
TGF-β: transforming growth factor-β
TP53: tumor protein 53
TUSON: tumor suppressor and oncogene
UCK2: uridine-cytidine kinase 2
WGCNA: weighted gene co-expression network analysis
WG: whole genome
WGS: whole-genome sequencing
WO: Washout
WRN: Werner syndrome helicase
XIST: X-inactive-specific transcript
