# Supplementary material for: Modeling specific aneuploidies: from karyotype manipulations to biological insights
Source: Chromosome Res. 2023 Aug 29;31(3):25. doi: 10.1007/s10577-023-09735-7 (PMC10462580; doi:10.1007/s10577-023-09735-7)
Supplement: Supplementary file 1 — (PDF 139 kb) [file 10577_2023_9735_MOESM1_ESM.pdf]

Modeling specific aneuploidies: from karyotype manipulations to biological insights

My Anh Truong*, Paula Cané-Gasull*, Susanne M. A. Lens#
Oncode Institute and Center for Molecular Medicine, University Medical Center Utrecht, Universiteitsweg 100, 3584 CG Utrecht, The Netherlands

*Equal contribution
#Corresponding author.
Email: s.m.a.lens@umcutrecht.nl

Supplementary information

Supplemental table 1: Overview of the cell models generated by MMCT and trisomic mouse embryos after crossings between mice harboring Robertsonian chromosomes ……………………………………………………………………………………………..*page 2*
Supplemental table 2: Overview of cell lines and mouse strains with CRISPR-Cas9-mediated chromosome fusions………...*page 3*
Supplemental table 3: Comprehensive overview of current approaches to induce whole and arm-level chromosome loss in mammalian cells ………..……*page 4*

Supplemental table 1. Overview of the cell models generated by MMCT and trisomic mouse embryos after crossings between mice harboring Robertsonian chromosomes

Approach	Models	Resulting trisomies	References
MMCT	hTERT-RPE1<br>*(hTERT immortalized retinal pigment epithelial)*	Ts3; Ts5; Ts12; Ts21	Passerini et al. 2016
	DLD1 *(colorectal cancer)*	Ts13; Ts7; Ts3	Phillips et al. 2001
	hTERT-HME<br>*(hTERT immortalized human mammary epithelial)*	Ts3	
	HE-35 (*human embryonic fibroblasts)*	Ts8	Nawata et al. 2011
	HCT116 *(colorectal cancer)*	Ts3; Ts5; Ts3+5	Haugen et al. 2008
	HCT116 *(colorectal cancer)*	Ts8; Ts13; Ts18; Ts21	Vasudevan et al. 2020
Crossing of mice with Robertsonian translocation	mESCs + whole mouse	Ts1; Ts13; Ts16; Ts19	Williams et al. 2008

hTERT, human telomerase reverse transcriptase; MMCT, Microcell-mediated chromosome transfer; mESC, mouse embryonic stem cell; Ts, Trisomy

Of note, a larger collection of human, rat, and mouse cancer cells containing an additional human chromosome has been generated by MMCT to map human tumor-supressor genes, and some of these earlier models already revealed effects of specific chromosomal gains in cancer (see main text; Uejima et al. 1995; Matsuda et al. 1997). These cell lines have been reviewed elsewhere (Kugoh et al. 2016) and are not included in this table.

Supplemental table 2. Overview of cell lines and mouse strains with CRISPR-Cas9-mediated chromosome fusions.

Type of fused chromosome	Cell type	Resulted fusion chromosomes	Viable heterozygous F1 pups*[1]	Viable homozygous F2 pups*[2]	References
Telocentric chromosome	ha-ESCs	Chr1+2	No	-	Zhang et al. 2022
		Chr4+5	Yes	Yes	
Robertsonian-like metacentric chromosome	AG-haESCs (sperm-like stem cells)	Rob(1;13)	Yes	Yes	Wang et al. 2022
		Rob(2; 6)	Yes	Not tested	
		Rob(2; 5)	Yes	Not tested	
		Rob(2; 11)	Yes	Yes	
		Rob(2; 18)	Yes	Not tested	
		Rob(5; 17)	Yes	Yes	
		Rob(7; 19)	Yes	Not tested	
		Rob(10;13)	Yes	Not tested	
		Rob(X;2), dup(14)	No	-	
		Rob(1;13).Rob(2;9)	Not tested	-	
		Rob(1;13).Rob(2;9).Rob(7;19)	Not tested	-	

AG-haESCs, androgenic haploid embryonic stem cells; haESCs, haploid embryonic stem cell; Rob, Robertsonian chromosome; dup, duplicated; *[1] embryos generated via oocytes injection of sperm-like nuclei carrying one fused chromosome; *[2] embryos generated via crossing of heterozygous F1 adults.

Supplemental table 3. Comprehensive overview of current approaches to induce whole and arm-level chromosome loss in mammalian cells

**Approach**			**Genome editing tool**	**Species**	**Model system**	**Targeted chromosome**	**Resulting aneuploidy**		**Selection method**	**References**
							**Type**	**Verification method**		
**Cre/*loxP*-mediated chromosome elimination**			Cre/*loxP*	mouse	mESC-somatic cell hybrids	6, 11, 12	whole	karyotyping, FISH painting	FACS	Matsumura et al. 2007
			Cre/*loxP*	mouse	mESC-somatic cell hybrids	6, 11, 12, 17	whole, segmental	karyotyping, FISH painting	FACS	Otsuji et al. 2008
			Cre/*loxP*	mouse	tetraploid MEFs	9, 10, 12, 14	whole, segmental	karyotyping, WGS	FACS	Thomas et al. 2018
			Cre/*loxP*	mouse	lymphocytes	4, 9, 10, 11, 14	whole, segmental	karyotyping, FACS	FACS	Zhu et al. 2010
			Cre/*loxP*	mouse	embryos	Y	whole	dot blot assay	-	Lewandoski and Martin 1997
			Cre/*loxP*	mouse	embryos	2	whole	FISH, qPCR	-	Grégoire and Kmita 2008
			Cre/*loxP*	human	HeLa	21	whole	FISH	HSV-TK	Sato et al. 2017
			Cre/*loxP*	human	DS iPSCs	21	whole	karyotyping, FISH	HSV-TK	Wakita et al. 2022
**Nuclease-mediated chromosome elimination**	**Truncation or fragmentation**	**Arm DSB(s)**	CRISPR/Cas9	mouse	mESCs	h14	whole, segmental	PCR genotyping, FISH, WGS	-	Zuo et al. 2017
			CRISPR/Cas9	mouse	mESCs	Y	whole, segmental or arm	FISH, qPCR	-	Adikusuma et al. 2017
			CRISPR/Cas9	mouse	mESCs	Y	whole	karyotyping FISH painting, WGS	-	Zuo et al. 2017
			CRISPR/Cas9	mouse	embryo brain	Y	whole	FISH painting	-	Zuo et al. 2017
			CRISPR/Cas9	mouse	zygotes	Y	whole	karyotyping, FISH painting, WGS	-	Zuo et al. 2017
			CRISPR/Cas9	mouse	zygotes	X	whole	karyotyping, FISH, WGS	-	Zuo et al. 2017
			CRISPR/Cas9	mouse	zygotes	2, 17	whole, segmental	WGS	-	Papathanasiou et al. 2021

**Nuclease-mediated chromosome elimination**	**Truncation or fragmentation**	**Arm DSB(s)**	CRISPR/Cas9	human	HT29	7	whole, structural changes (rearrangements)	FISH painting	-	Zuo et al. 2017
			CRISPR/Cas9	human	DS iPSCs	21	whole	FISH	-	Zuo et al. 2017
			CRISPR/Cas9	human	hTERT-RPE1	2p, 5q, 6q	arm, segmental, structural changes (rearrangements, fragmentation)	scWGS	-	Leibowitz et al. 2021
			CRISPR/Cas9	human	T cells	7q	segmental	scRNA-seq	-	Nahmad et al. 2022
			CRISPR/Cas9	human	zygotes	X	segmental	SNP array	-	Turocy et al. 2022
		**Centromeric or pericentromeric DSB(s)**	CRISPR/Cas9	mouse	mESCs	Y	whole	FISH, qPCR	-	Adikusuma et al. 2017
			CRISPR/Cas9	mouse	MB49	Y	whole	RT-qPCR	-	Abdel-Hafiz et al. 2023
			CRISPR/Cas9	mouse	hematopoietic stem cells	Y	whole	karyotyping, FISH	-	Sano et al. 2022
			CRISPR/Cas9	mouse	zygotes	Y	whole, arm	qPCR	-	Adikusuma et al. 2017
			CRISPR/Cas9	human	A2058	1q, 7p, 8q	arm	karyotyping, WGS	-	Girish et al. 2023
			CRISPR/Cas9	human	A2780	1q	arm	karyotyping, WGS	HSV-TK, FACS	Girish et al. 2023
			CRISPR/Cas9	human	AGS	1q	arm	WGS	HSV-TK, FACS	Girish et al. 2023
			CRISPR/Cas9	human	MCF10A	1q	arm	WGS	-	Girish et al. 2023
			CRISPR/Cas9	human	HCT116	8q	arm	WGS	-	Girish et al. 2023
			CRISPR/Cas9	human	T cells	14q	arm	FISH, ddPCR, scRNA-seq	-	Nahmad et al. 2022
			CRISPR/Cas9	human	zygotes, embryos	6q	whole, arm	SNP array	-	Zuccaro et al. 2020
			CRISPR/Cas9	human	zygotes	16, X	whole, arm, structural changes (fragmentation)	SNP array	-	Turocy et al. 2022
		**Centromere-proximal and -distal DSB(s)**	TALEN	human	MCF10A	8p12-8p23	segmental	SNP array	-	Cai et al. 2016
			CRISPR/Cas9	human	SKNSH	11q13-11q25	segmental	karyotyping, WGS	-	Eleveld et al. 2021
			CRISPR/Cas9	human	NMB	6q22-6q27	segmental	WGS	-	Eleveld et al. 2021

**Nuclease-mediated chromosome elimination**	**Telomere incorporation**	CRISPR/Cas9	hamster	CHO-K1	h21q	arm	karyotyping, FISH	Fcy::Fur, hisD	Uno et al. 2017
		CRISPR/Cas9	human	AALE	3p	arm	karyotyping, WGS	pac	Taylor et al. 2018
		CRISPR/Cas9	human	AALE	8p	arm	WGS	pac	Shih et al. 2023
		CRISPR/Cas9	human	AGS	1q	arm	karyotyping, WGS	pac	Girish et al. 2023
		CRISPR/Cas9	human	MCF10A	1q	arm	WGS	pac	Girish et al. 2023

AALE, human lung epithelial cell line; A2058, human melanoma cell line; A2780, human ovarian cancer cell line; AGS, human gastric cancer cell line; CHO-K1, Chinese hamster ovary-K1 cell line; ddPCR, digital droplet PCR; DS iPSCs, Down syndrome induced pluripotent stem cells; DSB, DNA double stranded break; FACS, fluorescence-activated cell sorting; Fcy::Fur, cytosine deaminase and uracil phosphoribosyl-transferase fusion gene; FISH, fluorescence in situ hybridization; HCT116, human colorectal cancer cell line; HeLa, human cervical cancer cell line; HisD, L-histidinol dihydrochloride gene; HR, homologous recombination; HSV-TK, herpes simplex virus thymidine kinase gene; HT29, human colorectal cancer cell line; MB49, mouse bladder cancer cell line; MCF10A, human mammary epithelial cell line; MEF, mouse embryonic fibroblasts; mESC, mouse embryonic stem cells; NMB, human neuroblastoma cell line; pac, puromycin N-acetyltransferase gene; qPCR, quantitative polymerase chain reaction; hTERT-RPE1, hTERT immortalized retinal pigment epithelial cell line; RT-qPCR, quantitative reverse transcription PCR; scRNA-seq, single-cell RNA sequencing; scWGS, single-cell whole genome sequencing; SKNSH, human neuroblastoma cell line; SNP array, single nucleotide polymorphism array; TALEN, transcription activator-like effector nuclease; WGS, whole genome sequencing; ZFN, zinc finger nuclease
